# DNA Methylation of the *ABO* Promoter Underlies Loss of *ABO* Allelic Expression in a Significant Proportion of Leukemic Patients

**DOI:** 10.1371/journal.pone.0004788

**Published:** 2009-03-10

**Authors:** Tina Bianco-Miotto, Damian J. Hussey, Tanya K. Day, Denise S. O'Keefe, Alexander Dobrovic

**Affiliations:** 1 Department of Haematology-Oncology and University of Adelaide, Discipline of Medicine, The Queen Elizabeth Hospital, Woodville, South Australia, Australia; 2 Dame Roma Mitchell Cancer Research Laboratories, Discipline of Medicine, The University of Adelaide, Hanson Institute, Adelaide, South Australia, Australia; 3 Department of Surgery, Flinders University, Flinders Medical Centre, Bedford Park, South Australia, Australia; 4 Department of Urology, University of Pittsburgh School of Medicine, Pittsburgh, Pennsylvania, United States of America; 5 Department of Pathology, Peter MacCallum Cancer Centre, Melbourne, Victoria, Australia; 6 Department of Pathology, University of Melbourne, Melbourne, Victoria, Australia; Deutsches Krebsforschungszentrum, Germany

## Abstract

**Background:**

Loss of A, B and H antigens from the red blood cells of patients with myeloid malignancies is a frequent occurrence. Previously, we have reported alterations in ABH antigens on the red blood cells of 55% of patients with myeloid malignancies.

**Methodology/Principal Findings:**

To determine the underlying molecular mechanisms of this loss, we assessed *ABO* allelic expression in 21 patients with ABH antigen loss previously identified by flow cytometric analysis as well as an additional 7 patients detected with ABH antigen changes by serology. When assessing *ABO* mRNA allelic expression, 6/12 (50%) patients with ABH antigen loss detected by flow cytometry and 5/7 (71%) of the patients with ABH antigen loss detected by serology had a corresponding *ABO* mRNA allelic loss of expression. We examined the *ABO* locus for copy number and DNA methylation alterations in 21 patients, 11 with loss of expression of one or both *ABO* alleles, and 10 patients with no detectable allelic loss of *ABO* mRNA expression. No loss of heterozygosity (LOH) at the *ABO* locus was observed in these patients. However in 8/11 (73%) patients with loss of *ABO* allelic expression, the *ABO* promoter was methylated compared with 2/10 (20%) of patients with no *ABO* allelic expression loss (*P* = 0.03).

**Conclusions/Significance:**

We have found that loss of ABH antigens in patients with hematological malignancies is associated with a corresponding loss of *ABO* allelic expression in a significant proportion of patients. Loss of *ABO* allelic expression was strongly associated with DNA methylation of the *ABO* promoter.

## Introduction

ABH antigens are carbohydrate structures present on the surface of red blood cells (RBCs) and platelets, as well as endothelial and epithelial cells. The antigens are generated by the stepwise addition of monosaccharides to protein or lipid core structures. Two glycosyltransferase genes catalyze the final steps of ABH antigen synthesis in RBCs. The precursor H antigen is determined by a fucosyltransferase coded for by *FUT1* [1]. The A and B glycosyltransferases, which add different monosaccharides to the precursor H antigen, are encoded by separate alleles of the *ABO* gene [2], [3]; the A glycosyltransferase which adds N-acetylgalactosamine to give the A antigen, and the B glycosyltransferase which adds galactose to give the B antigen. There are numerous weaker alleles of A and B coding for less active glycosyltransferases, the most common of which is *A^2^* [4]. The O allele is a null allele which is transcribed but is enzymatically inactive [3].

Alteration of ABH antigens in hematological malignancy was first reported by van Loghem *et al* [5] who described very weak A antigen expression on the RBCs of an acute myeloid leukemia (AML) patient, who had previously shown normal A antigen expression. Loss of A, B, or H antigens from the surface of RBCs has since then been a recurrent observation in transfusion laboratories dealing with hematological malignancy patients [6]–[8].

We previously described the use of a flow cytometric method for the sensitive detection of alterations of A, B and H antigens on RBCs [8]. Fifty-five percent (16/29) of patients with myeloid malignancies of blood group A, B, or AB had a detectable population of RBCs with decreased expression of A or B antigens compared with no detectable changes in 127 normal A, B, and AB individuals. Loss of H was detected in 21% (6/28) of group O patients compared with no changes in 51 normal O individuals.

Possible mechanisms for inactivation of *ABO* include allelic loss (loss of heterozygosity–LOH), mutation (loss of function) and silencing by DNA methylation. Loss of ABH antigens from tumor tissue is frequently seen in solid tumors including carcinomas of the buccal epithelium, stomach, colon, lung, ovary, prostate, bladder, and breast [9]–[18], and is associated with poor prognosis, high tumor grade and increased metastatic potential [9], [19]–[23]. Previous studies have found that loss of ABH antigens in solid tumors is associated with LOH [24]–[26].

The *ABO* promoter region is rich in CpG dinucleotides [27], [28] and previous analysis of this region in several human carcinoma cell lines and cancers has shown that DNA methylation of the *ABO* promoter region was inversely correlated with gene expression [25], [26], [29]. We set out to determine whether LOH and/or DNA methylation of *ABO* was responsible for ABH antigen alterations in patients with hematological malignancy.

## Materials and Methods

### Patient samples

The patients analyzed in this study presented to the Haematology-Oncology Department at The Queen Elizabeth Hospital during the period 1996–2000 with acute myeloid leukemia (AML), myelodysplastic syndrome (MDS) or myeloproliferative disorders (MPD) including chronic myeloid leukemia (CML). Twenty-one of the patient specimens analyzed were previously described in an analysis of ABH antigens by flow cytometry [8]. Seven additional patients were identified by serology as having loss of ABH antigens. Archival peripheral blood stem cell (PBSC) and bone marrow (BM) samples from breast cancer patients were used as controls, as well as peripheral blood mononuclear cells (PBMNC) from anonymous voluntary blood donors. For the leukemic patient samples, either bone marrow aspirates or peripheral blood, all samples were taken as part of routine clinical care and were surplus to diagnostic needs. The use of patient samples followed a protocol approved by the Human Research Ethics Committee of The Queen Elizabeth Hospital. Mononuclear cells were prepared from all patient specimens using Ficoll-Paque (Pharmacia, Uppsala, Sweden).

### Cell lines and 5-aza-2′-deoxycytidine treatments

Human leukemia cell lines EM-2, HEL, HL-60, K-562, KCL-22, JURKAT and RAJI were grown in RPMI 1640 with 10% fetal bovine serum (FBS), penicillin and streptomycin. Cells were maintained in a humid atmosphere containing 5% CO_2_ at 37°C. For 5-aza-2′-deoxycytidine (5-AZA) (Sigma, St Louis, MO) treatments, 10^6^ leukaemia cells were seeded in flasks and serum starved in medium supplemented with 0.1% FBS for 48 h prior to treatment. Following this, the medium was changed to include 10% FBS and cells were treated with 5-AZA (1 µM, 2 µM or vehicle - ultra pure water) daily for 3 days. Twenty-four hours after the final treatment, the media was removed, cells were washed with PBS and fresh media was added. Cells were allowed to recover for 24 h and were then harvested at 48, 72 and 96 h post treatment. RNA and DNA were isolated as outlined below, however, if there were less than 10^4^ cells after treatment due to extensive cell death by 5-AZA treatment, the cells were lysed with 0.3% Nonidet P40, 20 U RNAsin, 0.01 M DTT [30] and the supernatant was placed in TriPure for RNA extraction while the cell nuclei were bisulfite modified.

### RNA and DNA isolation

RNA was isolated with TriPure (Sigma) and genomic DNA was extracted by proteinase K/SDS treatment.[31] RNA was reverse transcribed using Moloney Murine Leukemia Virus reverse transcriptase (Invitrogen, Carlsbad, CA) according to the manufacturer's instructions. Genomic DNA was bisulfite modified as described previously [32], [33].

### PCR amplification

PCR reactions were performed in a volume of 50 µl and included 0.5 U HotStarTaq polymerase (Qiagen, Hilden, Germany), 2 mM MgCl_2_, 0.2 mM of each dNTP, 10 µM of each primer, 100 ng of bisulfite modified DNA or 2 µl of cDNA in the supplied buffer (Qiagen).

### *ABO* allelic expression analysis

**Figure 1:**
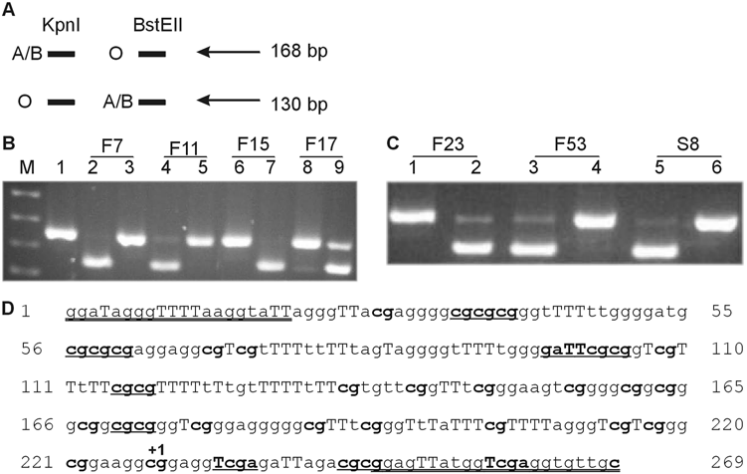
Loss of *A* expression by RT-PCR and restriction enzyme digestion. (A) Schematic representation of *ABO* allelic expression analysis. *Kpn*I digestion results in a 130 bp band if the O allele is present and no digestion of the A or B allele. *BstE*II digestion results in a 130 bp band if the A or B allele is present and no digestion of the O allele. (B) Lane M is the pUC19/*Hpa*II marker while lane 1 is the uncut *ABO* RT-PCR product. Lanes 2, 4, 6 and 8 are digested with *Kpn*I while lanes 3, 5, 7 and 9 are digested with *BstE*II. Lanes 2 and 3 are from cDNA of patient F7, lanes 4 and 5 from F11, lanes 6 and 7 from F15 and lanes 8 and 9 from F17. F7 and F11 are *AO* patients with loss of the A allele, F17 is an *AO* patient with no loss of *ABO* allelic expression. Patient F15 has an *A^1^A^2^* genotype, hence no cutting with *Kpn*I was expected. (C) Lanes 1, 3 and 5 are *ABO* RT-PCR product digested with *Kpn*I while lanes 2, 4 and 6 are digests with *BstE*II. Lanes 1 and 2 are from cDNA of patient F23, an *A^2^B* genotype, hence no cutting with *Kpn*I was expected. Lanes 3 and 4 are F53, an *A^1^O^1^* patient with loss of *A* at the mRNA level. Lanes 5 and 6 are S8, which is a patient with an *A^1^O^1^* genotype with loss of *A* allelic expression. (D) The *ABO* CpG island promoter region assessed for methylation. The methylated and bisulfite modified sequence is shown and the primer sequences are double underlined. The capital Ts identify thymines that are a result of bisulfite modification of cytosines and the CpGs are shown in bold. The start of transcription is marked with +1. The different restriction enzymes used for assessing methylation by digestion are as follows: eight *BstU*I sites (cg/cg), two *Taq*I (T/cga) sites (however one is found in the primer and hence will cut regardless of methylation status), one *Hinf*I (g/aTTc) site. Regions 161–173 and 198–210 harbor Sp1 sites [55].

*ABO* genotypes were determined as described previously [8]. The relative allelic expression of the *ABO* alleles in heterozygous patients was determined by restriction digestion of RT-PCR products. cDNA was amplified with primers ABO x5/6F (5′-caaaggtgctgacaccgtgtagga-3′) and ABO x6/7R (5′-ggaaagccacgtatttcttgatggc-3′) for *AO^1^* or *BO^1^* genotypes with the following PCR conditions 5 cycles of (30 s at 96°C, 60 s at 70°C - 1°C per cycle, 60 s at 72°C) followed by 35 cycles of (30 s at 96°C, 60 s at 65°C, 60 s at 72°C). The 168 bp PCR product was then digested with *BstE*II and *Kpn*I. Digestion with *Kpn*I was indicative of *O^1^* allele expression while digestion with *BstE*II was indicative of *A* or *B* allelic expression (Figure 1A). For genotypes which did not include the *O^1^* allele (for example *AB* and *A^1^A^2^* genotypes), the primers ABO x6/7F (5′-tgccatcaagaaatactgtgctttc-3′) and ABO R (5′-ctcgatgccgttggcctggtcga-3′) were used to amplify a 529 bp PCR product with the following PCR conditions: 40 cycles of (30 s at 96°C, 60 s at 68°C, 60 s at 72°C). Digestion of this PCR product with *Pvu*II indicated *A^2^* allelic expression and digestion with *Alu*I indicated *B* allelic expression. The primers and PCR conditions for the 377 bp reference gene were PBGD x1F (5′-ctttccaagcggagccatgtctgg-3′) and PBGD x6/7R (5′-catgagggttttcccgcttgcaga-3′), conditions 33–35 cycles of (30 s at 96°C, 60 s at 68°C, 60 s at 72°C).

### DNA methylation analysis

Methylation independent PCR (MIP) primers for *ABO* were designed to amplify bisulfite modified sequences regardless of methylation status[34] and are ABO bisF (5′-ggatagggttttaaggtattagggTT-3′) and ABO bisR (5′-gcaacacctcgAccatAActcc-3′). The uppercase Ts or As in the primer sequences indicate the position of a non CpG cytosine. The 269 bp PCR product (‘ABO BIS’) was amplified from bisulfite modified DNA with the following PCR conditions: 10 cycles (60 s at 94°C, 45 s at 65°C - 1°C per cycle, 45 s at 72°C) followed by 35 cycles of (60 s at 94°C, 60 s at 55°C, 60 s at 72°C). The ABO BIS PCR products were analyzed by methylation sensitive - single strand conformation analysis (MS-SSCA) [33] and/or COBRA (combined bisulfite restriction analysis) [35] and/or melt curve analysis (MCA) [25], [36]. For MS-SSCA, the ABO BIS products were analyzed on 0.5× and/or 0.75× MDE gels (FMC, Rockland, ME) [33]. For COBRA, the ABO BIS products were digested with the following restriction enzymes: *BstU*I, *Hinf*I and *Taq*I (all New England Biolabs, Beverly, MA). The restriction enzymes only digested the PCR product if the cytosine within the restriction enzyme recognition sequence was methylated. For methylation analysis by MCA, the ABO BIS PCR reactions were performed in 20 µl reactions using a Rotorgene 3000 real time PCR machine (Corbett Research, Sydney, Australia). Each reaction consisted of 10 µl of 2× Quantitect Sybr Green real time PCR mix (Qiagen), 2 µl of each primer (5 µM stock), and 6 µl of bisulfite modified DNA. Reactions were heated to 95°C for 15 min, then subjected to 55 cycles of 95°C for 15 s, 56°C for 30 s and 72°C for 30 s (acquisition at this step). After a final incubation at 72°C for 4 min, the melting profile was obtained by 90 s of pre-melt conditioning at 60°C then heating the reactions from 60°C to 99°C and acquiring the fluorescence at each 0.5°C increment. The controls for the MCA were a methylated control, which was a lymphocyte DNA sample treated with the SssI methylase enzyme and an unmethylated control which was lymphocyte DNA.

## Results

### Loss of heterozygosity studies at the *ABO* locus

The different nucleotide substitutions characteristic of the major ABO alleles create allele specific restriction enzyme sites. The deleted G in the O^1^ allele creates a KpnI site while the A and B alleles have a BstEII site at cDNA position 261. The A and B alleles are best distinguished by a base variation at position 467 and cut with AluI and PvuII respectively [37]. Digestion of the PCR products allows for the discrimination of the A, B and O alleles and is the easiest and most efficient way of genotyping at the ABO locus as well as determination of LOH [8], [38]–[40]. As most patients were AO heterozygotes, it was only necessary to type at the KpnI/BstEII site. Genotyping of patients for ABO did not reveal any allele shifts indicative of LOH (data not shown). We therefore decided to determine whether the ABO locus underwent allelic expression changes.

### *ABO* allelic expression studies in patient samples

The relative allelic expression of the *ABO* alleles was determined by allele specific restriction digestion of RT-PCR products which spanned the *Kpn*I/*BstE*II site (Figure 1A) and another product which spanned the *Alu*I*/Pvu*II site. We previously used this methodology to show that BM specimens had predominant expression of either *A* or *B* alleles relative to the *O* allele, probably due to nonsense mediated decay of the *O* allele [41].

Analysis of *ABO* expression, as assessed by RT-PCR and flow cytometry is summarized in Table 1. Combining patients from our previous flow cytometric studies of ABH antigen expression on RBCs [8] with additional patients identified through serology studies, there were 28 patients. Of these, 20 had detectable alterations of their A or B and/or H antigens on RBCs (Table 1). Twenty-seven of the patient samples were heterozygous at the *ABO* locus, and thus informative for detecting allelic expression changes. Thirteen of the 27 (48%) patient samples had altered allelic expression, either loss or reduced expression of an allele or no expression of either *ABO* allele (Table 1; Figure 1B and 1C). When divided into loss of ABH antigens detected by flow cytometric analysis compared with serology: 6/12 (50%) of the loss of ABH samples determined by flow cytometric analysis had a corresponding loss of *ABO* allelic expression by RT-PCR analysis compared with 5/7 (71%) in the group of patients with ABH antigen loss detected by serology.

**Table 1:** *ABO* genotyping, ABH antigen status and *ABO* allelic expression.

ID	DIAGNOSIS	GENO	FLOW ANALYSIS	EXP
F7	AML M2	A^2^O^1^	no loss	*loss of A^2^*
F9	AML	A^1^O^1^	**LOSS of A** & **H**	*little A^1^*
F10	MDS	A^1^O^1^	**LOSS of A**	*loss of A^1^ & O*
F11	AML M1	A^1^O^1^	no loss	*loss of A^1^*
F14	AML M2	A^1^O^1^	no loss	no loss
F15	CML blast crisis	A^1^A^2^	no loss	no loss
F17	MDS	A^1^O^1^	no loss	no loss
F20	CML	A^1^O^1^	**LOSS of H**	no loss
F23	CML chronic	A^2^B	no loss	no loss
F24	AML	BO^1^	**LOSS of B**	no loss
F25	MDS	A^1^A^1^	**LOSS of A**	no loss
F26	AML M3	A^1^O^1^	no loss	no loss
F27	AML M4	A^1^O^1^	**LOSS of A** & **H**	*loss of A^1^*
F30	CML chronic	BO^1^	**LOSS of H**	no loss
F39	AML M4	A^2^O^1^	**LOSS of H**	*loss of A^2^*
F42	AML M3	BO^1^	no loss	no loss
F46	AML M3	A^1^O^1^	**LOSS of A**	no loss
F51	AML M4	A^1^O^1^	**LOSS of A**	no loss
F53	AML M1	A^1^O^1^	**LOSS of H**	*loss of A^1^*
F57	AML M7	BO^1^	**LOSS of H**	no loss
F60	CML blast crisis	A^1^A^2^	**LOSS of A**	*'93+'96–*

ID	DIAGNOSIS	GENO	SEROLOGY	EXP
S1	RAEB	A^2^O^1^	**weak A2 expression**	*loss A^2^*
S2	MDS	A^1^O^1^	**LOSS of A**	*loss A^1^*
S3	CML	A^2^O^1^	**LOSS of A**	no loss
S5	AML M2	A^1^O^1^	**mfr→only H antigen**	*loss of A^1^*
S6	AML M3	A^2^B	**mfr with anti-A**	*loss of A^2^*
S7	AML	BO^1^	**mfr with anti-B**	no loss
S8	AML M0	A^1^O^1^	**LOSS of A**	*loss of A^1^*

In the ID column a F prefix denotes patients analyzed by flow cytometry [8] while a S prefix denotes loss of ABH antigen patients as detected by serology. ‘GENO’ refers to ABO genotype and ‘EXP’ to ABO allelic expression. For SEROLOGY ‘mfr’ refers to a mixed field reaction. In the EXP column, which allele is lost is shown in italics and underline. For patient F60 there were 2 samples analyzed for ABO mRNA expression, one in 1993 and one in 1996. The 1993 sample ('93+) was positive for *ABO* expression however the 1996 sample ('96–) which was when the flow analysis was performed, was negative for *ABO* mRNA expression. The bold indicates the samples with loss of ABH antigens. F25 was an *A^1^A^1^* sample therefore determining allelic expression was not possible.

Using the *ABO* RT-PCR restriction enzyme digestion method, an *AO* heterozygote sample predominantly expresses the *A* allele and thus restriction enzyme digestion is predominantly seen with *Bst*EII and not *Kpn*I (eg. F17 in Figure 1B). However, loss of *ABO* allelic expression, usually loss of the *A* allele as this is more frequent in the population than the *B* allele, results in a shift with predominantly digestion with *Kpn*I and not *BstE*II (eg. F7, F11, F53 and S8 in Figure 1B and 1C). F9 and F27, which had loss of both A and H antigens by flow cytometric analysis, had little or no expression of the *A^1^* allele. F10 and F60, both with loss of A by flow analysis had no expression of any *ABO* allele but were positive for the reference gene *PBGD*. However, for F60, a sample taken 3 years prior was positive for *ABO* expression. Though there was no flow cytometry performed for that sample, it seems likely that loss of *ABO* occurred as the malignancy progressed from the chronic phase to the blast crisis of CML. Loss of *A* allelic expression was also seen in the leukemic cells of four other patients, F7, F11, F39 and F53 (Figure 1B and 1C). F39 and F53 samples showed loss of the H antigen by flow cytometric analysis and in Figure 1C the RT-PCR sample from patient F53 digested only with *Kpn*I and not *BstE*II (lanes 3 and 4) indicating that this *A^1^O^1^* patient only expressed the *O^1^* allele. F7 and F11 were the only samples with loss of *ABO* allelic expression (lack of digestion with *Bst*EII – Figure 1B; lanes 3 and 5) but with no detectable alterations in ABH antigens by flow cytometric analysis. This apparently discrepant result may be due to the population of cells sampled for flow analysis not being representative of the population of cells with loss of *A* allelic expression, or the malignant cells not being able to differentiate to RBCs. One *A^1^O^1^* patient (F51) with complete loss of A by flow cytometry still had *A^1^* allelic expression. Sequencing of exon 7 of this patient, which contains the majority of the *ABO* coding sequence, did not detect any mutations (data not shown), however, a mutation may be present elsewhere in the gene. The remaining patients with loss of the A allele, were those with serologically detectable abnormalities in ABH antigens, S1, S2, S5, S6 and S8 (lack of digestion with *Bst*EII - Figure 1C lane 6).

### *ABO* allelic expression studies in leukemic cell lines

**Figure 2:**
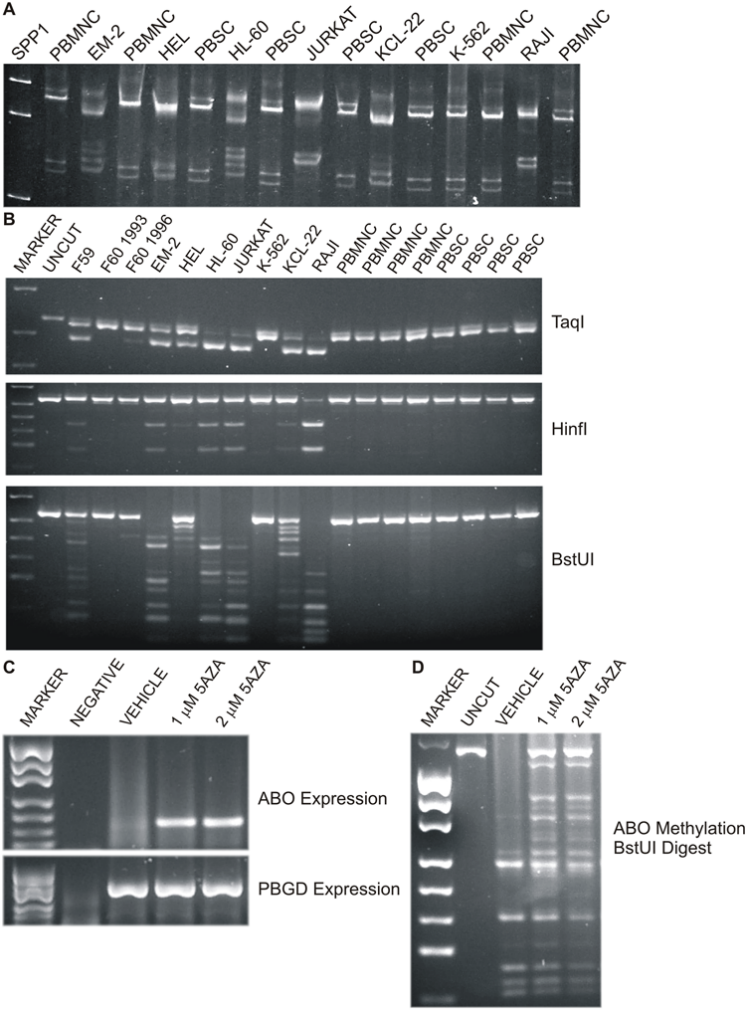
*ABO* promoter methylation in leukemic cell lines. (A) MS-SSCA analysis of the ABO BIS PCR products. PBMNC refers to peripheral blood mononuclear cells and PBSC to peripheral blood stem cells. These were used as unmethylated controls. It is clear from the SSCA gel that only the K-562 leukemic cell line is unmethylated as it has the same banding pattern as the PBMNC and PBSC. The other cell lines all have varying amounts of methylation as seen by the various banding patterns. The JURKAT and RAJI cell lines were hypermethylated, as seen by the dramatic shift of the bottom doublet of bands. (B) Restriction enzyme digests of the ABO BIS PCR products. Digestion with any of the restriction enzymes is indicative of methylation at that CpG site within the restriction enzyme recognition sequence. All the products will cut with *Taq*I since there is a *Taq*I site in the reverse primer. (C) *ABO* re-expression in the JURKAT cell line after 24 h treatment with 5-aza-2′-deoxycytidine treatment. On the gel, the NEGATIVE was an RT control (RNA only), the VEHICLE lane was JURKAT cells treated with ultra pure water, the following lanes are JURKAT cells treated with 1 µM or 2 µM of 5-aza-2′-deoxycytidine respectively showing *ABO* re-expression. *PBGD* is the reference gene. (D) The *ABO* promoter is demethylated in JURKAT cells after 5-aza-2′-deoxycytidine treatment. In the VEHICLE treated JURKAT cells there is no evidence of unmethylated ABO promoter which would be a band at the same size as the UNCUT sample. However, after treatment with 1 or 2 µM of 5-aza-2′-deoxycytidine the *ABO* promoter is unmethylated as evidenced by a band at the same size as the UNCUT sample.

Since LOH of the *ABO* locus was not observed in patient samples, we went on to assess DNA methylation of the *ABO* promoter. The region we examined spans the transcription start site and the bisulfite modified methylated sequence is shown in Figure 1D. We firstly investigated *ABO* promoter DNA methylation in leukemic cell lines since only 2/6 cell lines expressed *ABO* (Table 2), although it must be recognized that JURKAT and RAJI, which derive from lymphocytic leukemia would not be expected to express *ABO*. We investigated *ABO* DNA methylation in the leukemic cell lines by two methodologies, MS-SSCA [33] and COBRA [35]. From Figure 2A it is evident that only the K-562 cell line had the same banding pattern as the unmethylated PBMNC and PBSC samples. The remaining cell lines were methylated and lacked expression of *ABO*. The only cell line which expressed *ABO* but was methylated was the HEL cell line. Restriction enzyme digestion of the ABO BIS PCR products again showed that all the leukemic cell lines, except K-562 were methylated to various degrees (Figure 2B). HEL was the least methylated of the cell lines, perhaps indicative that only one of the *ABO* alleles was methylated and thus this cell line may still express *ABO* from the other allele.

**Table 2:** *ABO* genotyping, expression and methylation analysis of leukemic cell lines.

NAME	TYPE	GENO	EXP	METH	5-AZA
EM-2	Human CML in blast crisis	A^1^A^1^	−	M	+
HEL	Human erythroleukemia	O^1^O^1^	+	M	+
JURKAT	Human T cell leukemia	O^1^O^2^	−	M	+
K-562	Human CML in blast crisis	O^1^O^1^	+	U	+
KCL-22	Human CML in blast crisis	A^2^O^1^	−	M	+
RAJI	Human Burkitt lymphoma	O^1^O^1^	−	M	+

‘TYPE’ refers to the cell line type and origin, ‘GENO’ refers to the *ABO* genotype, ‘EXP’ to *ABO* mRNA expression and ‘METH’ to the *ABO* promoter CpG island methylation status as determined by MS-SSCA and COBRA (Figure 2). ‘5-AZA’ refers to *ABO* mRNA expression after the cell line was treated with 5-aza-2′-deoxyxytidine as outlined in the ‘Materials and Methods’. ‘−’ refers to negative *ABO* expression, ‘+’ refers to positive *ABO* expression, ‘U’ refers to unmethylated and ‘M’ to methylated *ABO* promoter region.

Since many of the leukemic cell lines were methylated and failed to express *ABO*, an attempt to re-express *ABO* was performed by treating the cells with the demethylating agent, 5-aza-2′-deoxycytidine (5-AZA). K-562 and HEL already expressed *ABO* and hence 5-AZA treatment resulted in no change (Table 2). The other cell lines EM-2, JURKAT, KCL-22 and RAJI were methylated and did not express *ABO* but after treatment with 5-AZA they all were demethylated and re-expressed *ABO*. The re-expression of *ABO* after treatment with 5-AZA in the JURKAT cell line is shown in Figure 2C as well as the corresponding demethylation at the *ABO* promoter (ie presence of uncut band in 5AZA treated JURKAT compared to vehicle treated - Figure 2D). Since *ABO* DNA promoter methylation was shown to be responsible for the silencing of this locus in the leukemic cell lines, we assessed whether *ABO* methylation was responsible for the loss of *ABO* allelic expression in the patient specimens.

### *ABO* methylation analysis in patients with hematological malignancies

The *ABO* promoter region was analyzed for methylation in the patient samples by three different methodologies: MS-SSCA, COBRA and MCA. Initially the samples were analyzed by MS-SSCA with 2 running conditions, [42], [43] followed by COBRA as a second method [35]. The restriction enzymes used to analyze *ABO* promoter methylation analysis were *Taq*I, *Hinf*I and *BstU*I. There were 34 CpGs in the ABO BIS PCR product (Figure 1D) and restriction enzyme digestion allowed analysis of 16 of these CpGs. No *ABO* promoter methylation was detected in the normal specimens, four PBMNC and four PBSC, despite a vast preponderance of non-expressing cells (Figure 2A and 2B).

**Figure 3:**
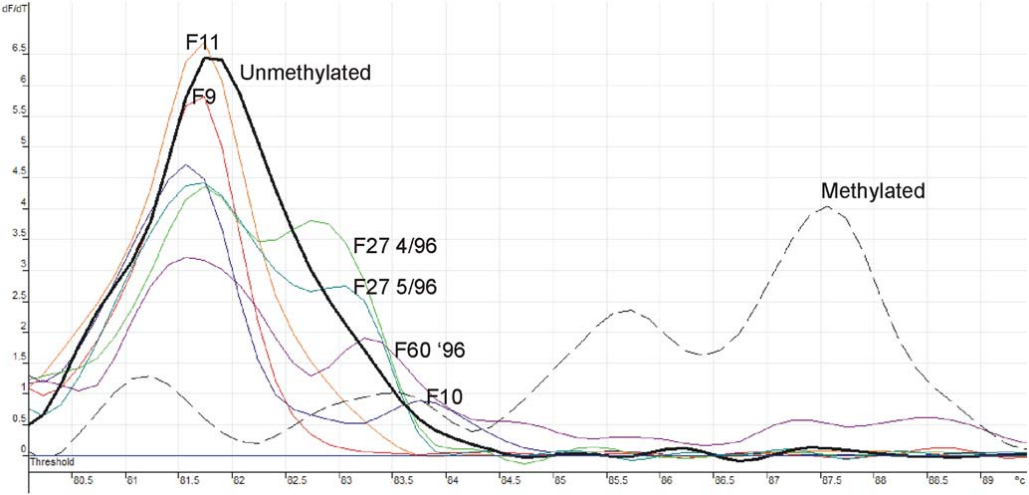
Melt curve analysis of the ABO BIS product in patients previously not shown to be methylated by MS-SSCA or COBRA. The methylated pattern is shown by a dashed line while the unmethylated with a dark black solid line. F9 and F11 both have loss of the *A^1^* allele but are unmethylated. F10 and F60 '96 are patient samples with no *ABO* allelic expression and show evidence of methylation by melt curve analysis. F27 is an *A^1^O^1^* patient with loss of the *A^1^* allele and methylation of 2 different samples, a month apart, which we had previously shown had increasing loss of ABH antigen loss [8] and both showed evidence of methylation.

Of the 21 patients analyzed for *ABO* promoter DNA methylation, 11 had loss of expression of one or both of the *ABO* alleles while 10 had no *ABO* allelic loss (Table 3). The *ABO* promoter was methylated in 8/11 (73%) of the patients with loss of *ABO* allelic expression compared with 2/10 (20%) of patients with no allelic loss (*P* = 0.03 by Fisher's exact test) (Table 3; Figure 3). Five of the 7 patients that were previously ascertained by serology and RT-PCR as having loss of A or B antigens were methylated at the *ABO* locus when analyzed by MS-SSCA and COBRA (patients with S prefixes, Table 3). COBRA was concordant with MS-SSCA except for sample S4 which was only methylated at one restriction site but unmethylated by MS-SSCA (data not shown), thus providing support for why more than one methodology was used. Methylation for 16 patients was additionally assessed using a more sensitive assay, MCA. After MCA, 6 additional patient samples, originally classified as unmethylated by MS-SSCA and COBRA, were found to be methylated (Figure 3), again providing further support for using more than one methodology. It is not surprising that MCA classified 6 additional patients as methylated as unlike MS-SSCA it does not rely on alterations in DNA strand conformations that result in obvious gel shifts, and unlike COBRA it does not rely on the CpG being assessed residing in a restriction enzyme site. MCA uses differences in melting temperature between methylated and unmethylated sequence, is relatively independent of the location of sequence differences within the PCR product, and is therefore able to detect differences in methylation at more sites than MS-SSCA or COBRA. Further, changes in melting curve profiles can result from even a single nucleotide change, therefore MCA can even detect methylation of one CpG site [36], [44].

**Table 3:** *ABO* methylation analysis in patient specimens with *ABO* allelic loss of expression.

ID	DIAGNOSIS	GENO	EXP	METH
F9	AML	A^1^O^1^	**little A^1^**	U
F10	MDS	A^1^O^1^	**loss of A^1^ & O**	**M**
F11	AML M1	A^1^O^1^	**loss of A^1^**	U
F15	CML blast crisis	A^1^A^2^	No loss	**M**
F17	MDS	A^1^O^1^	No loss	U
F20	CML	A^1^O^1^	No loss	U
F23	CML chronic	A^2^B	No loss	U
F24	AML	BO^1^	No loss	U
F27	AML M4	A^1^O^1^	**loss of A^1^**	**M**
F30	CML chronic	BO1	No loss	U
F39	AML M4	A^2^O^1^	**loss of A^2^**	**M**
F51	AML M4	A^1^O^1^	No loss	U
F53	AML M1	A^1^O^1^	**loss of A^1^**	**M**
F57	AML M7	BO^1^	No loss	U
F60	CML blast crisis	A^1^A^2^	**'93+'96–**	**M**

ID	DIAGNOSIS	GENO	EXP	METH
S1	RAEB	A^2^O^1^	**loss of A^2^**	U
S2	MDS	A^1^O^1^	**loss of A^1^**	**M**
S3	CML	A^2^O^1^	No loss	U
S5	AML M2	A^1^O^1^	**loss of A^1^**	**M**
S6	AML M3	A^2^B	**loss of A^2^**	**M**
S7	AML	BO^1^	no loss	**M**

In the ID column a F prefix denotes patients analyzed by flow cytometry while a S prefix denotes loss of ABH antigen patients as detected by serology. ‘GENO’ refers to *ABO* genotype, ‘METH’ to *ABO* promoter CpG island methylation assessed either by MS-SSCA, COBRA and/or melt curve analysis (MCA). ‘U’ unmethylated at the *ABO* promoter and ‘M’ is methylated at the *ABO* promoter. For patient F60 two samples were available for analysis; the 1993 ('93) sample was positive for *ABO* expression whereas the 1996 ('96) sample was negative for *ABO* expression.

## Discussion

Loss of ABH antigens in a subset of RBCs derived from a malignant stem cell is likely to be indicative of genetic or epigenetic changes that have occurred in the malignant stem cell. Malignant stem cells often retain the ability to differentiate along several lineages including the erythroid lineage [45]–[47]. RBCs that are deficient in A or B antigens have been reported to have decreased transferase activities, supporting the notion that loss of antigens reflects a change at the *ABO* locus and not at the cell surface or membrane precursors [48].

In this study, we examined whether loss of the A and B blood group antigens could be related to LOH at the *ABO* locus, differential expression of *ABO* alleles, or DNA methylation of the *ABO* promoter. It is surprising that no *ABO* LOH was observed as a substantial proportion of myeloid leukemias have deletions including the 9q34 region where *ABO* is located [49].

However, loss of mRNA expression of the corresponding *ABO* allele was seen for 11/19 (58%) of patients with loss of A and B antigens. Of these, 71% of samples shown to have loss by serology had detectable allelic loss at the mRNA expression level compared with 50% seen in patients with ABH antigen loss by flow analysis. The increased agreement in the samples with ABH antigen loss as determined by serology is not surprising since more than 50% of the cells need to have abnormal antigen expression to be readily detected by serology. The flow cytometric analysis can detect alterations in cell populations as low as 10%, and thus in these patient samples the larger normal population of cells would mask the expression changes of the smaller abnormal cell population. Therefore, it was not surprising that the mRNA analysis was more concordant with the loss of ABH antigen samples detected by serology compared with flow cytometry.

The 5-AZA treatment of the *ABO* negative leukemic cell lines indicated that *ABO* DNA methylation was associated with lack of *ABO* expression since demethylation of the *ABO* promoter resulted in re-expression of the gene. In the patients with *ABO* allelic loss, methylation of the *ABO* promoter was detected for 73% of the samples. Recently there has been a report in which *ABO* DNA methylation was found in leukemic patients [50] however since only the abstract was available we are not able to compare our results with this study. However, we show that DNA methylation is significantly associated with silencing of the *ABO* transcript in patients with hematological malignancies and that the *ABO* transcript can be re-expressed in leukemic cell lines by treating with a demethylating agent. DNA methylation of the *ABO* promoter would explain much of the reported loss of ABH antigens in patients with hematological malignancies [6].

There may of course be multiple mechanisms underlying the loss of ABH antigens in hematopoietic malignancy. It is intriguing to consider that *ABO* methylation may be part of a long range epigenetic silencing mechanism [51] leading to the co-ordinate silencing of a linked tumor suppressor gene may also be important in which case the alterations in *ABO* may be a sign post to a recurrent oncogenetic mechanism. This is supported by the observation that methylation in some cases is likely to lead to no cellular phenotype as was observed in OO individuals in which both alleles of the *ABO* locus are null or where the leukemic stem cells are unable to differentiate.

Additionally, since changes at the *ABO* locus have been associated with changes at other 9q34 loci, it is likely that *ABO* alterations are not the leukemia causing event but rather a marker of other events occurring at this chromosomal region. This is supported by the reported observation of individuals with decreases in both *ABO* and adenylate kinase (*AK1*) expression [52]–[54] in their leukemic cells. *AK1* is localized at 9q34.11, not too distant from *ABO* at 9q34.2. Further studies are needed to determine the importance of *ABO* alterations in leukemia and whether these are causative or an epiphenomenon.
